# Fishing filters flathead form and function in Tasmania

**DOI:** 10.1093/conphys/coag016

**Published:** 2026-03-24

**Authors:** Bradley E Howell

**Affiliations:** Department of Biology, Trent University, Peterborough, ON K9L 0G2, Canada

If you fish the same coastline intensively enough, the fish you catch today may not be the same as you would have caught a decade ago. Long-term fishing pressure can act as a filter, gradually favouring some traits over others. Understanding those trait shifts matters for management, because they can influence productivity, recovery potential, and long-term stability.

But can angling in heavily fished regions actually change the phenotype of fishes?

This question is especially relevant in Tasmania, where sand flathead (*Platycephalus bassensis*) support the state’s largest recreational fishery. In 2023 alone, more than one million sand flathead were caught, although 63% were released because they were under the legal size limit. In heavily fished southern regions, annual fishing mortality is estimated to remove roughly 93% of adult female sand flathead. These declines have already prompted tighter slot and bag limits. Meanwhile, northern Tasmania sees less fishing overall, partly due to its lower human population density and more exposed coastline, which makes boating access more difficult. This stark regional contrast provides a natural opportunity to test whether long-term harvest is associated with biological differences in wild fish.

**Figure 1:**
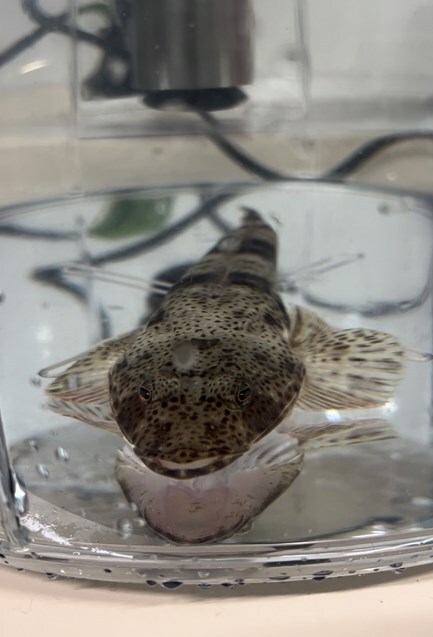
Sand flathead resting in a respirometry chamber. Image credit: Harriet Goodrich

Harriet Goodrich and her team from the University of Tasmania (Goodrich et al., 2026) compared sand flathead from heavily fished southern sites to individuals from lightly fished northern sites. They caught fish using hook-and-line gear similar to that used in the recreational fishery. They measured metabolic rate by tracking oxygen consumption while fish rested overnight in intermittent flow-through respirometry chambers (Fig. 1). They estimated age and growth using ear bones, whose growth rings reveal age in much the same way tree rings do. They assessed behaviour in a two-chamber “shuttle box” system, where they recorded how long fish took to explore a new area and whether they struck a bait placed in an unfamiliar space.

Two patterns stood out. Firstly, growth differed. Flathead from the south were consistently smaller than those from the north at any given age. This pattern is common in exploited fisheries, where repeated removal of large individuals shifts size structure toward smaller fish. Secondly, metabolism differed. Metabolic rate reflects how much energy an animal needs to maintain basic function, which we can estimate by how much oxygen it consumes. Flathead from the south used about 62% more energy on average than those from the north. This means that, even at rest, these fish are burning more energy to stay alive and operating closer to their energetic limits. Flathead from the south also showed a short-term rise in metabolism after capture that declined over time, suggesting stronger physiological reactivity or stress responses to disturbance. Behaviour told a different story. Tests of boldness and exploration showed no clear regional differences. Sand flathead are bottom-dwelling, site-attached ambush predators, so vulnerability to fishing capture may depend less on personality and more on physiological traits. Fish that burn more energy generally need to refuel more often, and that increased energetic demand may make them more likely to strike a bait or take risks while foraging. In this way, elevated metabolism alone could increase vulnerability to capture. The research team noted that environmental factors cannot be fully excluded from these findings. However, because the regions share similar habitats and temperatures, the differences observed are more likely linked to differences in fishing intensity.

This study highlights how conservation physiology can uncover changes that catch numbers alone cannot detect. If heavily fished populations of sand flathead shift toward smaller, higher metabolism individuals, their ability to cope with stressors such as ocean warming, food shortages or repeated fishing capture may be reduced. The message is clear: sustainable fisheries depend not only on maintaining abundance, but also on preserving the biological traits that support resilience.
